# Comparative assessment of three commercial kits and in house optimized PCR assays for GMO screening in food and feed

**DOI:** 10.1016/j.mex.2024.102878

**Published:** 2024-07-26

**Authors:** Daniela Verginelli, Cinzia Quarchioni, Katia Spinella, Davide La Rocca, Pamela Bonini, Cristiana Fusco, Marisa Misto, Stefania Peddis, Lorella Peroni, Ugo Marchesi

**Affiliations:** National Reference Laboratory for GM Food and Feed, GMO Unit, Istituto Zooprofilattico Sperimentale del Lazio e della Toscana “Mariano Aleandri”, Rome, Italy

**Keywords:** GMO, Screening elements, In-house verification, Multiplex assay, Commercial kits and in house optimized PCR assays for GMO screening

## Abstract

Screening strategies for GMO detection in food and feed are a crucial aspect in GMO testing laboratories for streamlining the analytical workflow and reducing turnaround time and costs. These strategies can be more or less complex or even be targeted according to the ingredients in the product, but whatever the choice, a good basic approach is generally based on the search for 35S promoter (P35S), nos-terminator (T-nos) and FMV promoter (P-FMV). In this study, we compare the singleplex real time PCR method for P35S, T-nos and P-FMV detection currently adopted by the Italian National Reference Laboratory for GM food and feed (NRL) with three commercial kits available on the market for giving a greater choice to consider the best approach suitable to the official control laboratories that are different from each other.

• The NRL optimized singleplex PCR methods and the three commercial kits fully respect all the validation parameters criteria according to the minimum performance requirements (MPR) of ENGL [1]
• Screening strategies for GMO detection in food and feed are a crucial aspect in GMO testing laboratories and being the commercial kits different from each other, the laboratory can choose the methods best suit their needs reducing turnaround time and costs.

## Graphical abstract

**Figure:**
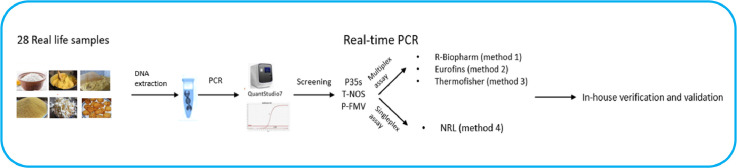

Specifications table

**Table:** 

Subject area:	Biochemistry, Genetics and Molecular Biology
More specific subject area:	Real-time PCR, screening assay
Name of your method:	Commercial kits and in house optimized PCR assays for GMO screening
Name and reference of original method:	Pauli U., Schouwey B., Hubner P., Brodmann P and Eugster A. Quantitative detection of genetically modified soybean and maize: Method evaluation in a Swiss ring trial. 2001. Mitt Lebensm Hyg. 92. 145–158; UNI EN ISO 21,569 2005/Amd.1:2013(E) Qualitative PCR method for detection of nopaline synthase terminator (QL-ELE-00–011) http://gmo-crl.jrc.ec.europa.eu/gmomethods/docs/QL-ELE-00–011.pdf; Horizontal methods for molecular biomarker analysis – Methods of analysis for the detection of genetically modified organisms and derived products – Part 5: Real-time PCR based screening method for the detection of the FMV promoter (P-FMV) DNA sequence; ISO/TS 21,569–5:2016 (2016). Qualitative PCR method for detection of Figwort Mosaic Virus 35S promoter (BLV L 00,00–148, 2014) (QL-ELE-00–015) http://gmo-crl.jrc.ec.europa.eu/gmomethods/docs/QL-ELE-00–015.pdf
Resource availability:	Details are provided in method section.

## Background

The current European Union (EU) legal framework regulating the presence of genetically modified organisms (GMO) in food and feed on the one hand provides for an unified authorization procedure, on the other imposes mandatory labeling and traceability for food and feed products containing authorized GM events, if more than 0.9 % GM-material is present in any food/feed ingredient (Reg CE 1829/2003 and Reg CE 1830/2003). According to this current legal framework within the EU market, one of the main tasks of GMO testing laboratories in official control is the analytical verification compliance with the labelling and traceability requirements for the presence of authorized genetically modified [2] events in food and feed products. This kind of verification is based on expensive and complex event-specific quantitative methods targeting the more than one hundred currently authorised GM events in addition to the constantly changing number of GM events with a pending/withdrawn authorization in the EU. It is however inconceivable, for logistical and economic reasons, to imagine performing such a large number of quantitative tests on each sample. Actually GMO testing is a complex and stepwise process consisting of consecutive analytical phases adopting PCR modules with increasing levels of specificity. After DNA extraction the first PCR step consists in verifying the presence of the reference gene/es related to the plant species appearing on the product label. Subsequent PCRs are carried out to search for screening elements and, in case of positivity, event-specific PCRs compatible with the screening profiles, and eventual quantifications are performed [[3], [4], [5], [6]]. Whatever the screening strategy adopted, that may differ from laboratory to laboratory, the approach followed by nearly all laboratories in the European Network of GMO Laboratories (ENGL) is the so called “matrix approach” [7], where GM events are matched against the screening elements sought on a table in matrix form showing the theoretical presence/absence of each screening element for each GM event. The analytical results obtained at the screening step on unknown samples can therefore be interpreted according to this matrix so as to rule out from subsequent analytical steps the search for GM events whose presence is incompatible with the obtained screening profile [8].

In the Italian National Reference Laboratory for GM food and feed (NRL) the screening strategy consists of several screening elements (P35S, T-nos, P-FMV, bar, pat, nptII, ctp2-CP4 epsps, TE9) [[9], [10], [11], [12], [13], [14], [15], [16], [17]]. The most common screening elements adopted by enforcement laboratories for revealing the presence of genetically modified organisms (GMO) in food and feed are typically P35S and T-nos. In addition P-FMV provides very useful further information to orientate following analyses. The screening elements are widely present in high number of authorized and unauthorized events, indeed the coverage (in%) on the authorized GM events in the EU including stacked events for P35S is 85.6 %, for T-nos is 83.8 and for P-FMV is 36.2 [18].

Considering how demanding it is for a laboratory to maintain in-house analytical methods under accreditation, for some time now some qualified companies have been offering standardized diagnostic kits for the different stages of GMO analysis, including the screening step. The aim of this study was to assess the technical performances of three commercially available PCR kits (number 1 and 2 are multiplex PCR for the three screening elements, the number 3 is based on duplex PCRs where the detection system for each screening element is coupled with the detection system for the corresponding natural) and internal real time singleplex PCR assays (number 4) routinely used in the laboratory (Table 1).

**Table 1:** Overview of Real‐time PCR kits evaluated in the study.

Number	Name of kit	Manufacturer	Real-time PCR assay	Target screening elements	Internal postive control	declared Limit of detection
1	4plex 35S/NOS/FMV/IAC^a^	R-Biopharm	triplex	35S/NOS/FMV	YES	≤ 5 DNA copies
2	GMOScreen RT 35S/NOS/FMV IPC^b^	Eurofins GeneScan	triplex	35S/NOS/FMV	YES	≤ 10 DNA copies
3	TaqMan™ GMO Screening Kit P35S/TNOS/ P34S-FMV^c^	Thermo Scientific	duplex with natural donor	35S/CaMV NOS/A.Tumefaciens P34S/FMV	YES	≤ 5 DNA copies
4	Internal laboratory method	Italian national reference laboratory	singleplex	P35S T- nos PFMV	NA	≤ 5 DNA copies

ᵃ35S is detected in FAM channel, NOS in Cy5 channel and FMV in ROX channel.

ᵇ35S is detected in FAM channel, NOS in JOE channel and FMV in TAMRA channel.

ᶜ35S, TNOS, P34S are detected in FAM channel and CaMV, A. tumefaciens, FMV are detected in VIC channel NA means not applicable.

## Method details

### Experimental design

**Fig. 1:**
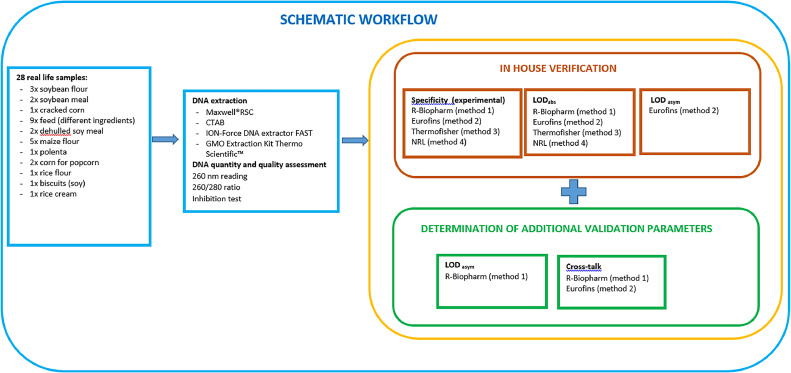
Schematic workflow representing the experimental design of the study. All samples were extracted in duplicate and analysed in two PCR replicates.

Twenty-eight samples were analyzed in parallel with three different commercial multiplex real-time PCR test kits for qualitative detection of the three screening elements P35S, T-nos and P-FMV and with the corresponding singleplex assays adopted and accredited by the Italian NRL for GM food and feed. In order to assess the performance of commercial multiplex PCR (mpPCR) qualitative kits, the validation parameters, if already described in the kit validation report, were verified or, if absent, were determined in the laboratory according to the in-house validation procedure (in line with the requirements set out in the ENGL Guidelines) [19]. The experimental design is illustrated in Fig. 1, we briefly described a practical approach for an in-house verification and validation of qualitative multiplex PCR methods. Each performance parameter is determined according to the guidelines reported by Grohmann et al. [19].

#### Notes and observations

Several real-time PCR-based screening methods are described and reported in specific method databases (e.g. EURL GMFF GMOMETHODS, EUginius) [18,20] and in scientific literature, including different versions of methods capable of detecting P35S, Tnos and P-FMV [[21], [22], [23]]. In many cases, such screening methods are implemented in their original form, as described in the references, but most of the time these protocols are optimised and adapted by the laboratory to make it possible to perform different tests at the same time in the same working session.

### Certified reference materials and real life samples

Twenty-eight real life samples, including processed food matrices containing soy, maize, rice, sugar beet, cotton or flax, were selected. Description and composition of each sample are listed in Table S1. The Certified Reference Materials (CRMs) MON89034 100 % maize (AOCS 0906 E2), T45 100 % canola (AOCS 0208-A), GA21 100 % maize (AOCS 0407-B), MON89788 100 % soybean (AOCS 0906-B), MON40–3–2 10 % soybean (ERM®- BF410ep) were purchased from the American Oil Chemists’ Society (AOCS, Urbana, Illinois, Urbana, USA) or from the European Commission's Joint Research Centre (JRC) and used as positive control samples in the analyses.

### Genetically modified screening elements detection methods

Three different commercial kits for GMO screening were assessed (Table 1). Two of them (method 1 and 2) consisted of a single multiplex real-time PCR assay targeting the same screening elements while the third one (method 3) consisted of a proprietary DNA extraction kit associated with three multiplex real-time PCR assays, as detailed below. All three kits have in common that they detect the three most widespread GMO screening elements: P35S, tNOS and P-FMV. Method 1 is a ready-to-use commercial kit (R-Biopharm: SureFood® GMO SCREEN 4plex 35S/NOS/FMV/IAC (Cat. No: S2126)) based on a multiplex assay for a qualitative detection of CaMV P35S, T-nos and P-FMV. Method 2 is another ready-to-use commercial kit (Eurofins GeneScan: GMOScreen RT 35S/NOS/FMV IPC (Cat. No: 5,421,220,301)) where the targets are detected by a real-time PCR multiplex assay. Method 3 is a ready-to-use commercial kit (ThermoScientific: TaqMan™ GMO Screening Kit P35S/TNOS/ P34S-FMV (Cat. No: 4,466,334)) made up of a DNA extraction kit followed by three real-time PCR duplex assays to simultaneously detect each of the three screening targets together with an endogenous sequence of its donor organism: 1) P35S/Cauliflower mosaic virus; 2) T-nos/Agrobacterium tumefaciens; 3) P34S/figwort mosaic virus using three different master mixes. In addition plant DNA as positive Control is amplified. All the commercial kits contain an internal positive control (IPC) to rule out the presence of inhibitors in the sample. The corresponding internal methods (method 4) targeting the three screening elements have long been within the scope of our laboratory's ISO 17,025 accreditation, and regularly used for routine analysis. These assays were optimized experimentally, modifying PCR reagents and, furthermore the PCR performance was assessed as reported by Woll et al. [24]. The qualitative method to detect P35S developed in laboratory, consists in a modified version of that previously described by Pauli et al. [9]: a different forward primer, designed to skip a single nucleotide polymorphism (SNP) affecting the amplification efficiency in the detection of P35S in DAS1507 maize event (DAS-Ø15Ø7–1). This qualitative method was validated by an in house evaluation and a collaborative study as reported by Gatto et al. [10]. The qualitative methods to detect T-nos and P-FMV are derived from the specific European Technical Specification (PD CEN/TS 16,707:2014) suitably modified using a different master mix and a different thermal profile [25]. The verification of PCR performance for the qualitative singleplex assays adopted by the Italian NRL was performed experimentally to assess the equivalence of the real-time PCR reagents used to replace those used in the validated method [26]. Practicability for T-nos and P-FMV has been checked on soybean MON40–3–2 10 % (ERM®- BF410ep) and maize MON89034 100 % (AOCS 0906 E2) using the master mix for the original validation QuantiTect Multiplex PCR NoROX Kit (Qiagen Gmbh, Germany) and the alternative master mix 1X TaqMan Universal Master Mix (Applied Biosystems, Foster City, CA).

#### Notes and observations

To further reduce the analytical workload, a number of commercial suppliers have started offering basic GMO screening kits that can detect the previously mentioned three elements. This can actually be an excellent alternative as long as the kit has comparable performance to the original methods. To verify the adoptability of these alternative solutions in the present study, three commercial kits, together with the corresponding internal methods already accredited by the testing laboratory, were verified in-house and, where necessary, additional validation parameters were determined, for the detection of P35S, Tnos and P-FMV on twenty-eight real life samples containing different ingredients. The performance parameters taken into account according to the ENGL guidelines [18] were Specificity, Absolut Limit of detection (LOD_abs_), Asymmetric LOD (LOD_asym_) and Cross-talk. The R-Biopharm (method 1) and Eurofins GenScan (method 2) mpPCR qualitative assays tested were designed from the providers in order to detect simultaneously P35S, T-nos and P-FMV in three different fluorescence channels. Differently, Thermo Fisher ScientificThermofisher’ kit (method 3) proposes an alternative approach for finding the three screening targets on the basis of the following considerations. Since the 35S promoter is originally derived from the Cauliflower Mosaic Virus (CaMV), the NOS terminator from tumour-inducing (Ti) plasmids of Agrobacterium tumefaciens and the FMV promoter from the figwort virus, these regulatory elements, if detected in the sample, could derive from a natural infection of the plants used for the preparation of the analysed foodstuff.

To reduce the chance of false positive results from these naturally occurring microbes, Thermo Fisher Scientific kit was designed as three duplex PCR assays, each of which detecting the GM element (P35S or T-nos or P-FMV) and the corresponding genomic sequence specific for its donor organism (Cauliflower mosaic virus, A. tumefaciens and figwort virus).

The internal laboratory assays (method 4), derived from already published methods detecting the screening elements considered in this study, have been further optimised to make their experimental conditions compatible with those already in use for other methods employed in the laboratory's diagnostic routine (e.g. using the same master mix and thus the same amplification profile). The singleplex assay for P35S was validated in a collaborative study involving 17 laboratories 10, while the T-nos and P-FMV assays were verified experimentally in order to assess the equivalence of real-time PCR reagent used. In addition, the three methods have a long history of accreditation at the national reference laboratory that conducted the tests in this study.

### DNA extraction

For all tests performed in this study, with the exception of ThermoScientific TaqMan™ GMO Screening Kit, DNA extraction was carried out in two replicates from 2 g of real life samples and 200 mg of certified Reference Material (CRM) used as positive control with the exception of CRM soybean MON89788 and CRM soybean MON40–3–2 where the intake had to be reduced down to 150 mg to help resuspension in lysis buffer. ION-Force DNA extractor FAST (©Generon S.p.A, Italy), CTAB method with minor modifications on buffer volume and, RSC PureFood GMO performed DNA extraction by using Maxwell®RSC Instrument (Promega Madison, WI, USA) as nucleic acid purification platform, according to the manufacturer's instructions. For testing with ThermoScientific: TaqMan™ GMO Screening Kit, upon specific request of the supplier, DNA was extracted by GMO Extraction Kit Thermo Scientific™ (Thermo Fisher Scientific Waltham, MA, USA), from 10 g of material according to the manufacturer's instructions.

### DNA yield and quality assessment

Concentration and purity of the extracted DNA were evaluated by spectrophotometric measurements (Eppendorf, Hamburg, DE) at 260 nm and 260/280 ratio according to the ISO 21,570:2005, Annex B [6] (on single stranded DNA) and following the instrument's operating manual.

### Real-time PCR assays

Real-Time PCR was performed on the QuantStudio7 (Life Technologies, Foster City, CA, USA) in a final volume of 25 μl with 100 ng of template DNA, using 1X TaqMan Universal Master Mix (Applied Biosystems, Foster City, CA). The thermal profile used for PCR reactions was: UNG pre-treatment of 2 min at 50 °C, initial activation of the polymerase for 10 min at 95 °C followed by 50 cycles of 95 °C for 15 s and 60 °C for 60 s. The baseline and the fluorescence threshold were automatically set up by the instrument software. Each commercial kit was used according to the manufacturer's instructions. References of the Italian NRL methods are indicated in the Table 2. The primes and probes for real time PCR were supplied by Eurofins genomics (Ebersberg, Germany).

**Table 2:** Italian NRL methods.

Gene name, primers and probes	final concentration	reference
**p35S (84bp amplicon)**		Gatto F and Paternò A, Report of the collaborative study for the validation of real time PCR methods for GMO screening, IZSLT, 2011 (Pauli et al., modified) https://www.izslt.it/crogm/pubblicazioni/ Pauli et al., “Quantitative detection of genetically modified soybean and maize: Method evaluation in a Swiss ring trial” (2001). Mitt. Lebensm. Hyg. vol. 92/2: 145–158
35S-F3	0.4 pmol/μl	
35S-R	0.4 pmol/μl	
35S-TMP	0.2 pmol/μl	
**tNOS (84bp amplicon)**		UNI EN ISO 21,569 2005/Amd.1:2013(E) Qualitative PCR method for detection of nopaline synthase terminator (GMOMETHODS: QL-ELE-00–011)
180 F	0.4 pmol/μl	
180 R	0.4 pmol/μl	
Tm-180	0.1 pmol/μl	
**pFMV (78bp amplicon)**		ISO/TS 21,569–5:2016(E) (GMOMETHODS: QL-ELE-00–015)
pFMV-F	0.34 pmol/μl	
pFMV-R	0.34 pmol/μl	
Probe pFMV	0.12 pmol/μl	
**LECTIN (LEC) GENE (74bp amplicon)**		GMOMETHODS: QT-TAX-GM-002
Lec-F	0.3 pmol/μl	
Lec-R	0.3 pmol/μl	
Lec-P	0.1 pmol/μl	
**HIGH-MOBILITY-GROUP (HMG) GENE (79bp amplicon)**		GMOMETHODS: QTTAX-ZM-002
MaiJ-F2	0.30 pmol/μl	
mhmg-rev	0.30 pmol/μl	
Mhmg-probe	0.18 pmol/μl	
**FIBER-SPECIFIC ACYL CARRIER PROTEIN (ACP1) GENE (76bp amplicon)**		GMOMETHODS: QT-TAX-GH-015
Acp1-F	0.15 pmol/μl	
Acp1-R	0.15 pmol/μl	
Acp1-P	0.05 pmol/μl	
**PHOSPHOLIPASE D (PLD) GENE (64bp amplicon)**		GMOMETHODS: QT-TAX-OS-017
KVM159	0.2 pmol/μl	
KVM160	0.2 pmol/μl	
TM013 probe	0.2 pmol/μl	
**GLUTAMINE SYNTHETASE (GS) GENE (121bp amplicon)**		GMOMETHODS: QT-TAX-BV-013
GLUA3-F	0.15 pmol/μl	
GLUA3-R	0.15 pmol/μl	
GLUD1-probe	0.1 pmol/μl	

### PCR inhibition test

The absence of PCR inhibitors in the DNA samples were evaluated according to the Annex 2 “Verification of analytical methods for GMO testing when implementing interlaboratory validated methods” JRC reported by Hougs et al., 2017 [26].

The inhibition test was performed in two dilution points of the taxon-specific reference systems. The amplification was performed in a final volume of 25 µl for the determination of quantification cycle (Cq) values at the two dilution levels (40 ng/ul and 10 ng/ul or undiluted and diluted 1:4) in two replicates of PCR. The difference between the measured Cq and the expected Cq was evaluated (ΔCq), considering the sample inhibited when ΔCq < 1.5 (Tab. S1).

### Specificity

The experimental specificity for all the qualitative methods was evaluated by the Italian NRL according to the Definition of Minimum Performance Requirements for Analytical Methods of GMO Testing part 2 JRC [1].The acceptance criterion used for the specificity parameter is that the PCR system developed should only produce amplification for the organisms for which the amplicon is expected to be detected. The experimental specificity was already evaluated by all the commercial kit providers and by the Italian NRL, several CRMs containing P35S and/or T-nos and/or P-FMV were tested. This parameter for the commercial kit Eurofins (method 2) was evaluated by the provider also in silico using BLAST. In this study, to investigate the capacity of a method to detect only the target analyte and discriminate all other targets we selected, and tested in duplicate, real life samples containing all, some, or no targets.

### Absolute lod verification

The determination of sensitivity was assessed measuring the limits of detection (LOD) defined as the lowest amount or concentration of analyte in a sample, which can be reliably detected, but not necessarily quantified. In the event that the LOD_abs_ was already determined and reported in the context of each method validation, the ENGL guidance document on multiplex PCR optionally suggests an intralaboratory verification [19]. Such verification was carried out by testing 10 PCR replicates at four serial dilutions contained approximately 20, 10, 5 and 1 copies/reaction of each target with the requirement to obtain amplification for all ten replicates. The LOD is considered the lowest copy number for which the ten PCRs are positive 20. The values were verified by using Quodata web application too (https://quodata.de/content/validation-qualitative-pcr-methods-single-laboratory/520fd1e35f2d601a4a96c7da9ddf4acc41a49000ad0c0166a2b580394b6443b2b41dba5d77c8776213c446b24b3477eed322f459984b808824d758e903002c7a253).

In addition for the method Eurofins (method 2) the LOD_abs_ is carried out with the verification kit for qualitative PCR Kit (Verification kit for qualitative PCR kits, ID2936) according to the manufacturer's instructions (data no shown).

### Asymmetric LOD determination

The LOD_asym_ is the lowest amount of an analyte in a sample that can be reliably detected, but not necessarily quantified, in the presence of high amount(s) of other target(s) in the multiplex PCR (mpPCR) assay [18]. All PCR replicates should provide positive results at LOD_asym_ conditions. For R-Biopharm kit (method 1) this performance parameter was estimated from scratch, as it was not included in the validation report of the supplier. For Eurofins GeneScan kit (method 2) an experimental verification of the data reported by the supplier was sufficient. Finally, for Thermofisher methods (method 3) and the NRL internal methods (method 4) this check was not applicable.

The concentration of DNA was checked by droplet digital PCR (ddPCR) as recommended by Grohman et al. [18] (data not shown). The verification of the LOD_asym_ for Eurofins GeneScan’ kit (method 2) was assessed in 10 PCR replicates for each target with 10 target copies/reaction in the presence of 5000 copies/reaction of all other targets (ratio 1/500).

The estimation of the LOD_asym_ for R-Biopharm (method 1) of each module is established by means of serial dilutions made from mixtures of the target DNA in excess of the other targets. Twelve PCR replicates at 20 copies/reaction of each target in the presence of 5000 copies/reaction of all other targets (ratio 1/250) were tested for each screening element. In the case of P35S and T-nos one or more PCR replicates were negative therefore twelve PCR replicates at 20 copies/reaction of P35S in the presence of 3000 copies/reaction of all other targets (ratio 1/150) and 25 copies/reaction of Tnos in the presence of 3000 copies/reaction of all other targets (ratio 1/120) were tested [19].

The CRMs T45 100 % canola (AOCS 0208-A) for P35S, GA21 100 % maize (AOCS 0407-B) for T-nos and MON89788 100 % soybean (AOCS 0906-B) for P-FMV were used as positive controls.

In addition for the method Eurofins (method 2) the LOD_asym_ is carried out with the verification kit for qualitative PCR Kit (Verification kit for qualitative PCR kits, ID2936) according to the manufacturer's instructions (data not shown).

### Crosstalk

The fluorescence signals generated during the amplification of different targets may bleed through between adjacent detection channels and superimpose each other and this may fake target sequence amplifications, resulting in false positives [19]. The experimental design of the cross-talk test is conducted in absence of the target and in 20.000 copies number for the other PCR module(s) in the respective channel(s) in three replicates per testing condition. The CRMs canola T45 100 % (AOCS 0208-A) for P35S; maize GA21 100 % (AOCS 0407-B) for t-NOS; soybean MON89788 100 % (AOCS 0906-B) for P-FMV were used as positive control. Acceptance Criteria: no cross-talk should be detected.

### DNA yield and quality assessment

The presence of inhibitors were investigated and the DNA extracted from 28 real life samples with different methods resulted no inhibited (Tab. S1). The DNA purity based on the average absorbance ratio 260/280 nm was close to the optimum value of 1.8 for almost all of samples. Samples analysed with the ThermoScientific GMO Screening kit (method 3) and extracted using the specific kit (GMO Extraction Kit Thermo Scientific) provided by Thermofisher, showed a lower DNA yield (sometimes <40 ng/ul) and a lower Cq of endogenous gene compared to other extraction kits.

### Experimental specificity

The Cq values obtained by testing 16 target and 12 non-target samples are summarized in Table 3A. The specificity was assessed measuring the number of false positive and the number of false negative samples (Table 3B). On the whole the verified results obtained for the detected screening elements are in accordance with the expected results. In only two expected negative samples weak signal (>38 Cq) were detected in one of the two PCR replicates.

**Table 3A:** Specificity test for the multiplex real-time PCR assay and NRL' assays.

				Verified											
				P35S				T-nos				P-FMV			
Number samples	Real life samples	GM event detected	Screening element expected	R-Biopharm	Eurofins GeneScan	Thermo Fisher	NRL	R-Biopharm	Eurofins GeneScan	Thermo Fisher	NRL	R-Biopharm	Eurofins GeneScan	Thermo Fisher	NRL
**1**	soybean flour (GM soy labelled)	MON40–3–2, MON87701, MON89788	P35S, T-nos, P-FMV	25.83±0.06	26.17±0.10	21.51±0.13	27.42±0.10	24.85±0.01	26.95±0.01	22.47±0.09	27.36±0.06	26.40±0.04	29.98±0.41	22.93±0.1	27.24±0.08
**2**	soybean flour (GM soy labelled)	MON40–3–2, MON87701, MON89789	P35S, T-nos, P-FMV	25.82±0.22	25.58±0.18	21.09±0.01	27.38±0.10	24.79±0.11	26.49±0.15	21.93±0.13	27.06±0.13	26.81±0.00	29.38±0.01	22.91±0.001	27.48±0.17
**3**	soybean meal (GM soy labelled)	A5547–127, A2704, MON40–3–2, MON87708, MON89788	P35S, T-nos, P-FMV	27.70±0.10	27.37±0.19	23.37±0.024	28.98±0.05	27.32±0.09	29.94±0.40	26.41±0.29	31.19±0.007	25.69±0.06	27.79±0.26	22.28±0.004	26.13±0.03
**4**	feed (GM soy and maize labelled; cotton)	GM soy and maize labelled, MON87460 spiked sample	P35S, T-nos, P-FMV	25.19±0.14	25.09±0.03	25.57±0.13	25.4 ± 0.14	24.34±0.09	26.04±0.13	26.38±0.02	29.25±0.07	25.55±0.12	28.29±0.32	25.76±0.22	26.24±0.19
**5**	feed (GM soy labelled; maize)	DAS1507, GA21, MON89034, NK603, MIR162, MON810, MON88017, BT11	P35S, T-nos, P-FMV	25.48±0.25	25.82±0.18	28.97±0.003	29.65±0.21	24.23±0.22	26.43±0.17	30±0.04	32.85±0.07	25.87±0.14	29.22±0.32	25.91±0.08	26.50±0.79
**6**	feed (GM soy and maize labelled; flax)	GM soybean and maize labelled	P35S, T-nos, P-FMV	26.15±0.15	28.95±0.06	28.67±0.12	29.15±0.07	27.29±0.13	30.30±0.22	30.54±0.22	32.85±0.07	24.58±0.23	28.72±0.41	26.55±0.07	28.27±0.14
**7**	feed (GM soy and maize labelled; cotton; sugarbeet)	GM soybean and maize labelled	P35S, T-nos, P-FMV	27.16±0.30	26.52±0.07	28.92±0.29	27.85±0.21	27.68±0.28	27.50±0.13	29.95±0.4	30.1 ± 0.00	25.52±0.25	34.10±1.63	27.82±0.26	27.56±0.21
**8**	dehulled soy meal (GM soy labelled)	A2704, MON40–3–2, MON87708, MON89788	P35S, T-nos, P-FMV	26.93±0.15	27.69±0.09	22.86±0.09	29.015±0.09	27.87±0.11	29.71±0.12	25.11±0.001	30.59±0.007	25.30±0.01	28.61±0.05	22.33±0.03	27.56±0.21
**9**	dehulled soy meal (GM soy labelled)	A2704, MON40–3–2, MON87708, MON89789, DP305423	P35S, T-nos, P-FMV	26.28±0.06	27.08±0.23	25.83±0.26	27.45±0.07	27.50±0.10	29.26±0.013	26.74±0.41	30.7 ± 0.14	25.40±0.01	28.39±0.01	25.56±0.32	26.74±0.71
**10**	soy meal (GM labelled)	MON40–3–2, MON87708, MON89788	P35S, T-nos, P-FMV	25.91±0.86	26.79±0.25	21.84±0.2	27.86±0.007	26.55±0.60	27.64±0.15	22.56±0.31	28.13±0.05	26.61±0.67	29.63±0.18	23.71±0.16	28.37±0.22
**11**	maize flour (raw material)	MON40–3–2, MON87708, MON89789	P35S, T-nos, P-FMV	24.08±0.29	27.55±0.02	30.08±0.19	30.95±0.77	25.83±0.05	28.43±0.11	31.36±0.16	31.9 ± 0.5	25.22±0.02	29.38±0.13	26.24±0.07	25.44±0.19
**12**	polenta (maize)	Spiked sample Bt176	P35S	30.20±0.29	31.87±0.45	38.64±0.08	32.06±0.08	ND	ND	ND	ND	ND	ND	ND	ND
**13**	feed (GM soy and maize labelled; cotton; flax)	GM soybean and maize labelled	P35S, T-nos, P-FMV	26.91±0.01	28.93±0.15	27.84±0.24	26.75±0.21	27.92±0.03	29.88±0.26	29.02±0.01	29.15±0.35	27.37±0.14	33.47±0.22	27.6 ± 0.03	27.09±0.07
**14**	cracked corn (GM maize labelled)	MON810, MON89034, NK603	P35S, T-nos, P-FMV	28.74±0.28	31.04±0.02	28.19±0.85	31.31±0.021	30.71±0.14	32.33±0.26	28.46±1.01	31.7 ± 0.3	29.33±0.02	34.93±0.21	29.63±0.9	31.61±0.48
**15**	corn for popcorn	MON89034 NQ, NK603 NQ	P35S, T-nos, P-FMV	30.07±0.16	32.26±0.27	32.76±0.16	34.53±0.59	33.76±0.16	34.69±0.18	32.77±0.005	36.64±0.37	34.59±0.04	36.36±1.37	33.95±0.11	35.59±0.38
**16**	feed (GM soy and maize labelled, sugarbeet)	GM soybean and maize labelled	P35S, T-nos, P-FMV	23.47±0.02	26.44±0.34	27.55±0.001	27.75±0.21	25.18±0.10	27.63±0.18	28.89±0.06	29.3 ± 0.14	23.40±0.19	25.21±0.38	27.18±0.06	27.33±0.24
**17**	feed (soy)	not tested	Negative	ND	ND	ND	ND	ND	ND^1^	ND	ND	ND	ND	ND	ND
**18**	corn for popcorn	not tested	Negative	ND	ND	ND	ND	ND	ND	ND	ND	ND	ND	ND	ND
**19**	maize flour	not tested	Negative	ND	ND	ND	ND	ND	ND	ND	ND	ND	ND	ND	ND
**20**	maize flour	not tested	Negative	ND	ND	ND	ND	ND	ND	ND	ND	ND	ND	ND	ND
**21**	soybean flour	not tested	Negative	ND	ND	ND	ND	ND	ND	ND	ND	ND	ND	ND	ND
**22**	feed (maize, soy)	not tested	Negative	ND	ND	ND	ND	ND	ND	ND	ND	ND^1^	ND	ND	ND
**23**	rice flour	not tested	Negative	ND	ND	ND	ND	ND	ND	ND	ND	ND	ND	ND	ND
**24**	feed (maize, rice)	not tested	Negative	ND	ND	ND	ND	ND	ND	ND	ND	ND	ND	ND	ND
**25**	biscuits (soy)	not tested	Negative	ND	ND	ND	ND	ND	ND	ND	ND	ND	ND	ND	ND
**26**	rice cream	not tested	Negative	ND	ND	ND	ND	ND	ND	ND	ND	ND	ND	ND	ND
**27**	maize flour	not tested	Negative	ND	ND	ND	ND	ND	ND	ND	ND	ND	ND	ND	ND
**28**	maize flour	not tested	Negative	ND	ND	ND	ND	ND	ND	ND	ND	ND	ND	ND	ND
**POS CTR**	positive control kit	NA	P35S, T-nos, P-FMV	27.01	31.04	31.61	NA	25.47	31.91	32.38	NA	28.46	32.9	31.8	NA
**POS CTR**	positive control NRL (MON89034 100 % GM, AOCS 0906-E)	MON89034	P35S, T-nos, P-FMV	25.73	26.53	23.47	24.24	26.74	27.39	23.63	25.32	27.33	28.4	23.14	21.95

**NTC**	**NTC**	NA	Blank	ND	ND	ND	ND	ND	ND	ND	ND	ND	ND	ND	ND

For positive amplification signals, Cq mean value and standard deviation are indicated; NQ means not quantified (<LOQ), ND means not detected, NA means not applicable.

1 undetermined and Cq ≥38.

**Table 3B:** number of false positive samples and false negative samples obtained in the study.

			Verified							
			R-Biopharm		Eurofins GeneScan		Thermofisher		NRL	
Target	Expected		False positive samples	False negative samples	False positive samples	False negative samples	False positive samples	False negative samples	False positive samples	False negative samples
**P35S**	Positive	16	0	0	0	0	0	0	0	0
	Negative	12								
**T-nos**	Positive	15	0	0	0	0	0	0	0	0
	Negative	13								
**PFMV**	Positive	15	0	0	0	0	0	0	0	0
	Negative	13								

#### Notes and observation

The in-house verification data presented here show that all three multiplex real-time PCR assays and singleplex assays are fit for purpose for the reliable detection of GM food and feed. The specificity was evaluated in twenty-eight real life samples including matrices that are relevant for a testing laboratory e.g. soybean, maize, cotton, rice and sugar beet with different degree of processing as highly processed matrices such as biscuits and polenta, in order to have a wide range of the applicability in routine testing of these qualitative assays. To confirm the results, CRMs were used as positive controls.

The overall assessment of the commercial screening systems considered in this paper is rather favorable in terms of both applicability and practicability. The targets chosen are those most popular with GMO testing laboratories and the multiplex kit format facilitates the operator's work and reduces processing time.

Regarding specificity the results showed that all PCR methods performed appropriately in detecting the screening targets in all the samples (just for two PCR replicates a weak signal above 38 Cq was detected).

### Verification of absolute LOD

LOD_abs_ was verified for all methods tested. For each PCR method tested, an amplification signal was detected for all ten replicates at 5 target copies. The observed result was the same as expected for all methods (Table 4). Quodata elaborations confirmed the obtained results (Fig.S1).

**Table 4:** Estimation of the absolute limit of detection (LOD_abs_). The CRM used was MON89034 100 % maize (AOCS 0906-E2); NA means not ampliflied.

R-Biopharm (Method 1)		*declared LOD: 5 gen copies*												
	Cq					Cq					Cq			
GM copies number	20	10	5	1	GM copies number	20	10	5	1	GM copies number	20	10	5	1

LOD_abs_ P35S (FAM)	31.53	30.76	30.20	NA	LOD_abs_ T-nos (Cy5)	30.36	32.21	31.03	NA	LOD_abs_ PFMV (ROX)	29.62	30.55	30.88	NA
	29.95	29.65	30.80	NA		30.10	31.42	33.26	NA		29.13	30.78	30.52	NA
	30.28	30.54	30.99	38.06		30.85	30.92	32.93	NA		29.46	30.00	31.71	32.90
	29.38	29.24	29.99	NA		30.08	31.37	31.56	NA		29.30	30.97	31.28	32.77
	29.74	30.44	29.80	35.94		29.85	31.94	31.43	NA		28.89	30.76	29.95	NA
	29.09	29.68	29.62	34.66		30.77	32.10	31.97	NA		29.27	30.32	30.50	NA
	29.48	29.81	31.34	NA		30.60	30.10	32.74	NA		29.11	29.80	31.44	32.56
	29.12	29.60	29.10	NA		30.10	31.61	31.97	NA		29.20	29.65	30.58	NA
	29.17	29.52	30.15	NA		30.42	31.02	30.85	NA		29.34	29.74	30.75	NA
	28.92	29.29	29.07	35.68		29.84	30.79	34.62	32.23		29.72	29.69	31.03	NA
positive n.	10	10	10	4	positive n.	10	10	10	1	positive n.	10	10	10	3
%	100	100	100	40	%	100	100	100	10	%	100	100	100	30
Eurofins GeneScan (Method 2)	*declared LOD: 10 gen copies*													
	Cq					Cq					Cq			
GM copies number	20	10	5	1	GM copies number	20	10	5	1	GM copies number	20	10	5	1

LOD_abs_ P35S (FAM)	34.65	35.33	34.62	39.13	LOD_abs_ T-nos (JOE)	35.18	36.12	35.44	NA	LOD_abs_ PFMV (TAMRA)	34.57	35.66	36.32	NA
	33.96	34.84	35.39	38.55		34.56	35.13	36.88	40.66		34.66	35.38	36.94	NA
	33.71	35.12	35.62	38.56		34.94	35.72	36.82	38.82		34.35	35.79	36.96	36.98
	34.07	34.55	35.48	43.91		33.90	35.59	35.78	37.80		34.48	35.07	36.56	38.52
	34.54	35.48	34.98	NA		33.92	35.23	35.44	37.20		34.26	35.18	34.87	NA
	33.73	34.77	35.21	37.37		33.74	35.94	37.54	NA		34.06	35.02	36.07	NA
	33.55	34.99	36.78	NA		34.60	35.28	37.85	NA		34.54	34.99	37.11	38.29
	33.95	34.16	36.96	NA		35.27	35.67	36.89	NA		34.37	35.36	38.05	37.54
	34.07	35.04	35.85	NA		34.07	35.00	36.19	NA		34.62	35.51	36.31	NA
	33.86	34.31	35.67	NA		33.90	35.06	35.80	38.67		34.30	35.57	36.94	39.25
n. pos	10	10	10	5	n. pos	10	10	10	5	n. pos	10	10	10	5
%	100	100	100	50	%	100	100	100	50	%	100	100	100	50
ThermoScientific (Method 3)	*declared LOD: 5 gen copies*													
	Cq					Cq					Cq			
GM copies number	20	10	5	1	GM copies number	20	10	5	1	GM copies number	20	10	5	1

LOD_abs_ P35S (FAM)	34.66	35.56	36.52	39.02	LOD_abs_ T-nos (FAM)	32.81	33.32	34.17	NA	LOD_abs_ PFMV (FAM)	35.38	35.77	38.69	39.27
	35.46	35.30	36.64	40.08		32.64	33.74	34.85	NA		35.11	36.59	37.99	NA
	35.16	35.24	38.28	38.12		32.43	33.26	36.13	NA		35.03	37.23	37.43	39.70
	34.65	35.93	36.55	39.03		32.64	33.93	36.03	NA		35.08	37.05	36.40	38.22
	34.67	35.05	36.51	NA		32.24	33.81	34.52	36.44		34.98	36.60	37.40	39.19
	34.58	35.39	35.79	38.98		33.03	33.67	34.99	35.33		34.56	36.15	37.58	39.02
	34.69	35.35	37.41	38.18		33.85	33.98	34.78	NA		34.99	36.31	38.64	39.32
	34.37	35.38	37.06	38.19		33.15	33.87	34.66	38.53		35.98	36.42	37.36	39.14
	34.72	36.32	37.98	38.74		32.62	33.55	34.98	36.16		35.62	35.89	37.41	38.41
	34.62	35.94	36.53	37.80		33.17	33.30	35.17	35.63		37.00	36.61	38.55	39.27
n. pos	10	10	10	9	n. pos	10	10	10	5	n. pos	10	10	10	9
%	100	100	100	90	%	100	100	100	50	%	100	100	100	90
Italian NRL methods (Method 4)	*LOD P35S; T-nos; PFMV: 5 cp*													
	Cq					Cq					Cq			
GM copies number	20	10	5	1	GM copies number	20	10	5	1	GM copies number	20	10	5	1

LOD_abs_ P35S (FAM)	34.98	35.12	35.99	37.75	LOD_abs_ T-nos (FAM)	34.85	35.57	36.81	39.68	LOD_abs_ PFMV (FAM)	33.42	34.12	37.79	38.31
	33.99	35.13	36.60	38.69		34.30	36.88	36.89	38.13		33.62	34.88	34.89	35.82
	34.02	35.33	37.82	NA		35.26	36.28	37.21	NA		33.16	34.52	36.16	38.91
	34.03	35.50	36.89	37.53		35.22	36.80	38.50	39.87		33.60	34.80	37.74	37.57
	34.05	34.98	36.22	NA		34.80	36.18	36.89	NA		33.19	35.12	35.15	36.58
	33.87	36.09	36.78	38.63		35.24	37.72	36.36	NA		33.35	35.41	35.84	NA
	33.91	36.08	35.95	39.03		34.84	35.90	36.22	NA		33.97	35.45	35.74	37.39
	33.79	35.41	35.30	37.56		34.97	35.85	38.08	40.60		33.96	34.57	35.97	37.56
	34.06	34.65	37.33	38.64		34.80	36.50	38.97	37.87		34.14	35.17	36.82	36.89
	34.14	34.79	35.97	NA		35.35	35.36	37.53	37.77		33.64	34.95	35.34	38.69
n. pos	10	10	10	7	n. pos	10	10	10	6	n. pos	10	10	10	9
%	100	100	100	70	%	100	100	100	60	%	100	100	100	90
LOD_95 %_ values verified by using Quodata web application														
Screening kit	LOD													
	P35S	T-NOS	PFMV											

R-Biopharm (Method 1)	4.42	6.72	5.15											
Eurofins GeneScan (Method 2)	3.73	3.73	3.73											
ThermoScientific (Method 3)	1.3	3.73	1.3											
Italian NRL methods (Method 4)	2.44	3.06	1.3											

#### Notes and observation

Although absolute limit of detection (LOD_abs_) is indicated as optional in the “Guidance document on multiplex real-time PCR methods” [19] for the validation and verification of mpPCR this parameter were evaluated as well. The LOD_abs_ of the different commercial systems in multiplex format resulted having a perfect match with the three singleplex assays adopted by NRL (5 copies for all targets).

### Estimation of asymmetric LOD

The LOD_asym_ was determined per each target in presence of high amounts of the other targets as described by Grohmann et al. [19]. For R-Biopharm kit (method 1) a positive signal was detected for all twelve replicates with 20 copies of P-35S in a background of 3000 copies (1/150), 25 copies of Tnos in a background of 3000 copies (1/120) and 20 copies of P-FMV in a background of 5000 copies (1/250). For EurofinsGenScan (method 2) the LOD_asym_ verification a positive signal was detected in all ten replicates at 10 target copies in a background of 5000 non-target copies (1/500) (Table 5).

**Table 5:** Estimation of the asymmetric limit of detection (LOD_asym_). Positive control used were T45 100 % canola (AOCS 0208-A) for p35S; GA21 100 % maize (AOCS 0407-B) for tNOS and MON89788 100 % soybean (AOCS 0906-B) for pFMV.

R-Biopharm (Method 1)								
DETERMINATION OF LOD_asym_ R-Biopharm								
LOD_asym_ p35S (FAM) 20 copies / 1500 copies + 1500 copies (1/150)			LOD_asym_ tNOS (Cy5) 25 copies / 1500 copies + 1500 copies (1/120)			LOD_asym_ pFMV (ROX) 20 copies / 2500 copies + 2500 copies (1/250)		
Cq 20 copies T45 (p35S)	Cq Background 1500 copies GA21 (tNOS)	Cq Background 1500 copies MON89788 (pFMV)	Cq 25 copies GA21 (tNOS)	Cq Background 1500 copies T45 (p35S)	Cq Background 1500 copies MON89788 (pFMV)	Cq 20 copies MON89788 (pFMV)	Cq Background 2500 copies T45 (p35S)	Cq Background 2500 copies GA21 (tNOS)
33.46	26.86	26.90	32.62	27.05	26.56	30.08	25.26	30.35
35.75	26.61	26.70	32.76	27.08	26.60	30.37	25.40	30.70
33.88	26.51	26.51	31.75	26.99	26.56	30.66	25.34	30.97
32.76	26.51	26.55	32.69	26.90	26.47	31.33	25.08	31.79
35.29	26.42	26.39	36.13	26.60	26.46	30.24	25.09	30.61
34.22	26.60	26.57	32.20	26.89	26.37	30.37	25.06	30.73
36.45	26.64	26.48	31.13	26.67	26.42	31.01	25.08	31.52
33.80	26.36	26.44	31.95	26.84	26.41	30.52	24.95	30.91
33.14	26.75	26.35	36.58	26.97	26.51	30.58	25.22	30.94
38.90	26.54	26.58	34.52	26.84	26.47	30.53	25.10	30.89
35.17	26.44	26.49	32.95	26.75	26.45	30.27	25.16	30.61
34.58	26.55	26.53	34.31	27.15	26.91	31.06	25.31	31.55
**Eurofins-GeneScan (Method 2)**								

**VERIFICATION OF LOD_asym_ Eurofins**								

**LOD_asym_ p35S (FAM) 10 copies / 2500 copies + 2500 copies (1/500)**			**LOD_asym_ tNOS (JOE) 10 copies / 2500 copies + 2500 copies (1/500)**			**LOD_asym_ pFMV (TAMRA) 10 copies / 2500 copies + 2500 copies (1/500)**		
Cq 10 copies T45 (p35S)	Cq Background 2500 copies GA21 (tNOS)	Cq Background 2500 copies MON89788 (pFMV)	Cq 10 copies GA21 (tNOS)	Cq Background 2500 copies T45 (p35S)	Cq Background 2500 copies MON89788 (pFMV)	Cq 10 copies MON89788 (pFMV)	Cq Background 2500 copies T45 (p35S)	Cq Background 2500 copies GA21 (tNOS)

34.53	26.54	27.62	33.43	26.85	27.37	35.79	26.85	26.48
33.87	26.56	27.61	33.88	26.58	26.99	36.23	26.71	26.47
34.96	26.39	27.28	33.30	26.65	27.07	35.66	26.71	26.24
35.29	26.33	27.46	33.45	26.45	27.18	35.52	26.67	26.17
34.66	26.23	27.30	33.54	26.40	26.95	33.90	26.39	26.16
33.98	26.33	26.81	34.38	26.47	26.77	35.04	26.40	26.18
34.92	26.32	27.09	33.92	26.37	26.98	34.91	26.77	26.39
33.85	26.39	27.25	33.63	26.39	26.75	35.60	26.62	26.25
33.66	26.27	27.17	33.99	26.46	27.14	34.47	26.56	26.25
34.49	26.24	27.32	34.04	26.51	27.32	35.20	26.48	26.28

#### Notes and observation

Asymmetric LOD (LOD_asym_) was obviously considered for mpPCR systems only. This parameter showed a better performance for Eurofins GeneScan kit (method 2) than for R-Biopharm kit (method 1). This could have potential effects in the case of very unbalanced presence in the sample of the three screenings elements, however in the real life samples analysed in this study no differences could be detected.

### Crosstalk

Cross-talk evaluation was carried out for 3 PCR replicates as described in Method details. The R-Biopharm and Eurofins Gene scan kits did not cross-react and had no influence on sensitivity since no interfering fluorescence signals were found in the multiplex PCR tests (Table 6).

**Table 6:** Evaluation of cross-talk signals. Positive control used were T45 100 % canola (AOCS 0208-A) for P35S; GA21 100 % maize (AOCS 0407-B) for tNOS; MON89788 100 % soybean (AOCS 0906-B) for PFMV.

**CROSS-TALK R-Biopharm (method 1)**				**CROSS-TALK Eurofins GeneScan (method 2)**			
**P35S (FAM)**				**P35S (FAM)**			
Cq 0 copies/reaction T45 (P35S)	Cq 20,000 copies/reaction GA21 (t-NOS)	Cq 20,000 copies/reaction MON89788 (PFMV)	Cq IPC (VIC)	Cq 0 copies/reaction T45 (P35S)	Cq 20,000 copies/reaction GA21 (t-NOS)	Cq 20,000 copies/reaction MON89788 (PFMV)	Cq IPC (Cy5)
Undetermined	22.95	22.71	27.32	Undetermined	23.14	25.33	33.87
Undetermined	22.73	22.59	28.35	Undetermined	23.08	24.87	33.26
Undetermined	22.65	22.49	27.82	Undetermined	22.98	24.9	32.77

**t-NOS (Cy5)**				**t-NOS (JOE)**			

Cq 0 copies/reaction GA21(t-NOS)	Cq 20,000 copies/reaction T45 (P35S)	Cq 20,000 copies/reaction MON89788 (PFMV)	Cq IPC (VIC)	Cq 0 copies/reaction GA21 (t-NOS)	Cq 20,000 copies/reaction T45 (P35S)	Cq 20,000 copies/reaction MON89788 (PFMV)	Cq IPC (Cy5)
Undetermined	23.12	23.04	25.82	Undetermined	23.92	24.95	33.47
Undetermined	22.97	22.94	26.72	Undetermined	23.64	24.73	33.59
Undetermined	22.97	22.94	25.62	Undetermined	23.7	24.59	32.72

**PFMV (ROX)**				**PFMV (TAMRA)**			

Cq 0 copies/reaction MON89788 (PFMV)	Cq 20,000 copies/reaction T45 (P35S)	Cq 20,000 copies/reaction GA21 (t-NOS)	Cq IPC (VIC)	Cq 0 copies/reaction MON89788 (PFMV)	Cq 20,000 copies/reaction T45 (P35S)	Cq 20,000 copies/reaction GA21 (t-NOS)	Cq IPC (Cy5)

Undetermined	23.18	23.27	24.5	Undetermined	24.02	23.41	33.4
Undetermined	22.84	22.94	25.4	Undetermined	23.79	23.2	32.98
Undetermined	22.86	22.94	25.33	Undetermined	23.69	22.95	33.18

#### Notes and observation

Remaining with the two multiplex methods (method 1 and method 2), in both cases the validation reports provided by the manufacturers did not contain any crosstalk evaluation data. However, our study showed no interference between the different fluorescence detection channels [1], this parameter is evaluated and no crosstalk was observed in the mpPCR avoiding the risk of false positives.

## Conclusion

In GMO testing laboratories the screening strategy is a crucial step as it guides and optimises the, far more expensive and cumbersome, event-specific qualitative and quantitative PCR steps that may follow as a result of positivities. Here we presented a preliminary comparative evaluation of three commercial kits, together with the corresponding in-house optimized PCR tests, targeting the three screening elements sought by almost all GMO testing laboratories, namely P35S, T-nos and P-FMV. Although limited to a few screening elements, the application of the ENGL guidelines for the validation and verification of methods in singleplex and multiplex PCR provided data on the performance of such commercial solutions. The results confirm that the kits show high sensitivity, specificity, and practicability showing a substantial equivalence with the singleplex assays currently adopted by the Italian National Reference Laboratory for GM food and feed (NRL). The work done could be useful for all laboratories to evaluate the possibility of introducing commercial diagnostic kits for the screening phase of their workflow for GMO analyses to reduce and optimize workloads.

## Limitations

None.

## CRediT authorship contribution statement

**Daniela Verginelli:** Conceptualization, Methodology, Investigation, Writing – original draft, Writing – review & editing. **Cinzia Quarchioni:** Investigation. **Katia Spinella:** Visualization, Formal analysis, Writing – review & editing. **Davide La Rocca:** Visualization, Formal analysis, Writing – review & editing. **Pamela Bonini:** Investigation. **Cristiana Fusco:** Investigation. **Marisa Misto:** Investigation. **Stefania Peddis:** Investigation. **Lorella Peroni:** Investigation. **Ugo Marchesi:** Conceptualization, Writing – review & editing, Supervision.

## Declaration of competing interest

The authors declare that they have no known competing financial interests or personal relationships that could have appeared to influence the work reported in this paper.

## Data Availability

Data will be made available on request.

## Appendix Supplementary materials

Image, application 1

Image, application 2
