# Supplementary material for: Comparative assessment of three commercial kits and in house optimized PCR assays for GMO screening in food and feed
Source: MethodsX. 2024 Jul 26;13:102878. doi: 10.1016/j.mex.2024.102878 (PMC11345693; doi:10.1016/j.mex.2024.102878)
Supplement: Supplementary file 2 [file mmc2.docx]

Tab S1. DNA quality (A260/280) and inhibition test obtained for real life samples using spectrophotometric method

| **n.** | **Species and reference gene** | **Real life samples** | **NRL**  **EXTRACTION METHODS** | **purity** | **Inhibition test** | | | **THERMO SCIENTIFIC^TM^ EXTRACTION METHOD** | **purity** | **Inhibition test** | | |
| --- | --- | --- | --- | --- | --- | --- | --- | --- | --- | --- | --- | --- |
|  |  |  |  | **260/280 ratio** | **Cq**  **40 ng/ml** | **Cq**  **10 ng/ml** | **ΔCt** |  | **260/280 ratio** | **Cq**  **40 ng/ml or undiluted** | **Cq 10 ng/ml or diluted 1:4** | **ΔCt** |
| **1** | soybean (lectin) | soybean flour (GM soy labelled) | Maxwell®RSC | 1.84 | 25.9 | 27.91 | 2.05 | " | 1.46 | 20.57^a^ | 22.50 | 1.93 |
|  |  |  |  | 1.84 | 25.91 | 27.98 | 2.07 |  | 1.37 | 20.56^a^ | 22.57 | 2.01 |
| **2** | soybean (lectin) | soybean flour (GM soy labelled) | Maxwell®RSC | 1.86 | 25.94 | 27.89 | 1.95 | " | 1.51 | 20.20 | 22.16 | 1.96 |
|  |  |  |  | 1.86 | 25.90 | 27.94 | 2.04 |  | 1.51 | 20.35 | 22.31 | 1.96 |
| **3** | soybean (lectin) | soybean meal (GM soy labelled) | Maxwell®RSC | 1.87 | 26.14 | 28.18 | 2.04 | " | 0.78 | 21.12^a^ | 23.26 | 2.14 |
|  |  |  |  | 1.86 | 26.14 | 28.24 | 2.11 |  | 1.28 | 21.19^a^ | 23.22 | 2.03 |
| **4** | cotton (ACP1) | feed (GM soy and maize labelled; cotton) | CTAB | 1.84 | 26.40 | 28.60 | 2.20 | " | 1.82 | 28.66 | 31.79 | 3.13 |
|  |  |  |  | 1.85 | 26.10 | 28.20 | 2.10 |  | 1.93 | 28.65 | 31.53 | 2.88 |
| **5** | maize (HMG) | feed (GM soy labelled; maize) | Maxwell®RSC | 1.80 | 26.24 | 28.94 | 2.70 | " | 2.17 | 25.50 | 27.58 | 2.08 |
|  |  |  |  | 1.78 | 26.03 | 28.76 | 2.73 |  | 1.75 | 25.40 | 27.71 | 2.31 |
| **6** | maize (HMG) | feed (GM soy and maize labelled; flax) | CTAB | 1.72 | 27.90 | 30.10 | 2.20 | " | 1.68 | 26.06 | 28.14 | 2.09 |
|  |  |  |  | 1.70 | 28.00 | 30.20 | 2.20 |  | 1.60 | 25.87 | 27.97 | 2.10 |
| **7** | cotton (ACP1) | feed (GM soy and maize labelled; cotton; sugarbeet) | CTAB | 1.86 | 25.50 | 27.70 | 2.30 | " | 1.94 | 26.96^a^ | 29.12 | 2.16 |
|  |  |  |  | 1.87 | 25.40 | 28.60 | 3.20 |  | 1.78 | 26.25^a^ | 28.63 | 2.38 |
| **8** | soybean (lectin) | dehulled soy meal (GM soy labelled) | Maxwell®RSC | 1.89 | 27.09 | 29.13 | 2.05 | " | 1.20 | 21.09 | 23.34 | 2.25 |
|  |  |  |  | 1.88 | 26.89 | 28.98 | 2.09 |  | 1.19 | 21.19 | 23.40 | 2.21 |
| **9** | soybean (lectin) | dehulled soy meal (GM soy labelled) | Maxwell®RSC | 1.91 | 26.71 | 28.85 | 2.14 | " | 1.79 | 24.68 | 26.65 | 1.97 |
|  |  |  |  | 1.92 | 26.81 | 28.79 | 1.98 |  | 2.12 | 24.28 | 26.28 | 2.00 |
| **10** | soybean (lectin) | soybean meal (GM labelled) | Maxwell®RSC | 1.92 | 26.64 | 28.54 | 1.90 | " | 2.07 | 20.71 | 22.85 | 2.15 |
|  |  |  |  | 1.91 | 26.70 | 28.83 | 2.13 |  | 2.36 | 20.21 | 23.30 | 3.10 |
| **11** | maize (HMG) | maize flour (raw material) | CTAB | 1.90 | 23.10 | 25.40 | 2.20 | " | 1.81 | 25.70 | 28.07 | 2.37 |
|  |  |  |  | 1.88 | 23.90 | 26.10 | 2.20 |  | 1.76 | 25.98 | 28.25 | 2.27 |
| **12** | maize (HMG) | polenta | ION-Force DNA extractor FAST | 1.71 | 22.02 | 23.92 | 1.90 | " | 1.85 | 21.44 | 24.06 | 2.62 |
|  |  |  |  | 1.70 | 22.21 | 24.22 | 2.02 |  | 1.71 | 21.75 | 23.86 | 2.11 |
| **13** | cotton (ACP1) | feed (GM soy and maize labelled; cotton; flax) | CTAB | 1.85 | 27.90 | 30.00 | 2.10 | " | 1.80 | 26.25 | 28.68 | 2.44 |
|  |  |  |  | 1.87 | 28.20 | 29.80 | 1.60 |  | 1.79 | 26.46 | 28.84 | 2.38 |
| **14** | maize (HMG) | cracked corn (GM labelled) | Maxwell®RSC | 1.85 | 25.38 | 27.19 | 1.82 | " | 1.71 | 21.66 | 23.72 | 2.05 |
|  |  |  |  | 1.82 | 24.94 | 27.32 | 2.38 |  | 1.90 | 22.97 | 24.95 | 1.98 |
| **15** | maize (HMG) | corn for popcorn | Maxwell®RSC | 1.79 | 22.97 | 25.87 | 2.90 | " | 1.62 | 21.75 | 23.76 | 2.01 |
|  |  |  |  | 1.78 | 23.54 | 25.74 | 2.20 |  | 1.56 | 21.61 | 23.72 | 2.11 |
| **16** | sugarbeet  (GS) | feed (GM soy and maize labelled; sugarbeet) | CTAB | 1.84 | 36.20 | 37.70 | 1.50 | " | 1.67 | 27.48 | 29.46 | 1.98 |
|  |  |  |  | 1.84 | 36.30 | 38.40 | 2.10 |  | 1.61 | 27.27 | 29.62 | 2.35 |
| **17** | soybean (lectin) | feed (soy) | Maxwell®RSC | 1.81 | 26.89 | 28.96 | 2.07 | " | 1.83 | 25.31 | 27.54 | 2.23 |
|  |  |  |  | 1.80 | 26.85 | 28.87 | 2.02 |  | 1.86 | 25.43 | 27.84 | 2.41 |
| **18** | maize (HMG) | corn for popcorn | Maxwell®RSC | 1.71 | 22.83 | 24.83 | 2.01 | " | 1.75 | 22.03 | 24.18 | 2.15 |
|  |  |  |  | 1.73 | 23.40 | 25.38 | 1.97 |  | 1.74 | 22.10 | 24.52 | 2.42 |
| **19** | maize (HMG) | maize flour | Maxwell®RSC | 1.77 | 25.05 | 27.05 | 2.00 | " | 1.77 | 20.51 | 22.49 | 1.98 |
|  |  |  |  | 1.75 | 25.32 | 27.35 | 2.03 |  | 1.81 | 20.45 | 22.48 | 2.03 |
| **20** | maize (HMG) | maize flour | Maxwell®RSC | 1.77 | 24.49 | 26.52 | 2.03 | " | 1.80 | 21.05 | 23.61 | 2.56 |
|  |  |  |  | 1.79 | 24.03 | 26.59 | 2.56 |  | 1.77 | 22.37 | 24.76 | 2.39 |
| **21** | soybean (lectin) | soybean flour | Maxwell®RSC | 1.88 | 29.71 | 31.42 | 1.71 | " | 1.83 | 23.01^a^ | 24.86 | 1.85 |
|  |  |  |  | 1.88 | 29.09 | 31.42 | 1.71 |  | 2.14 | 22.98^a^ | 24.62 | 1.64 |
| **22** | maize (HMG) | feed (maize. soy) | Maxwell®RSC | 1.82 | 30.09 | 32.34 | 2.25 | " | 1.79 | 26.88 | 29.23 | 2.35 |
|  |  |  |  | 1.85 | 30.11 | 32.38 | 2.26 |  | 1.82 | 26.73 | 29.14 | 2.41 |
| **23** | rice (PLD) | rice flour | Maxwell®RSC | 1.69 | 24.72 | 26.80 | 2.08 | " | 1.67 | 25.91^a^ | 28.33 | 2.41 |
|  |  |  |  | 1.72 | 24.64 | 26.77 | 2.13 |  | 1.70 | 26.11^a^ | 29.02 | 2.91 |
| **24** | maize (HMG) | feed (maize; rice) | Maxwell®RSC | 1.73 | 24.87 | 26.99 | 2.12 | " | 1.79 | 28.96 | 31.09 | 2.13 |
|  |  |  |  | 1.76 | 24.85 | 26.87 | 2.01 |  | 1.83 | 29.21 | 31.15 | 1.94 |
| **25** | soybean (lectin) | biscuits (soy) | Maxwell®RSC | 1.81 | 27.99 | 29.40 | 1.41 | " | 2.06 | 33.17^a^ | 35.31 | 2.14 |
|  |  |  |  | 1.92 | 27.83 | 29.48 | 1.65 |  | 2.59 | 33.25^a^ | 35.61 | 2.36 |
| **26** | rice (PLD) | rice cream | Maxwell®RSC | 1.73 | 25.72 | 27.79 | 2.07 | " | 2.85 | 31.16^a^ | 32.99 | 1.83 |
|  |  |  |  | 1.74 | 25.71 | 28.03 | 2.32 |  | 2.82 | 31.28^a^ | 33.04 | 1.76 |
| **27** | maize (HMG) | maize flour | Maxwell®RSC | 1.80 | 24.74 | 26.75 | 2.01 | " | 1.78 | 23.58 | 25.69 | 2.11 |
|  |  |  |  | 1.88 | 24.82 | 26.85 | 2.03 |  | 1.78 | 23.64 | 25.93 | 2.29 |
| **28** | maize (HMG) | maize flour | Maxwell®RSC | 1.75 | 23.71 | 25.80 | 2.09 | " | 1.76 | 22.17 | 24.38 | 2.21 |
|  |  |  |  | 1.81 | 23.97 | 25.95 | 1.98 |  | 1.73 | 22.32 | 24.62 | 2.30 |
| ^a^ <40 ng/ml | |  |  |  |  |  |  |  |  |  |  |  |
