# Supplementary material for: Comparative assessment of three commercial kits and in house optimized PCR assays for GMO screening in food and feed
Source: MethodsX. 2024 Jul 26;13:102878. doi: 10.1016/j.mex.2024.102878 (PMC11345693; doi:10.1016/j.mex.2024.102878)
Supplement: Supplementary file 2 [file mmc1.docx]

FigS1. Calculation of $LOD_{95\%}$ based on the POD curve obtained with Quodata web application

R-Biopharm (Method 1)

LOD P35S

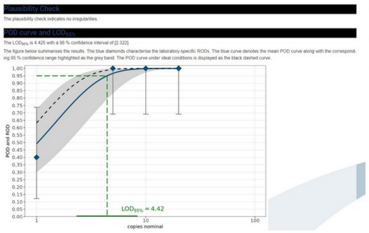

LOD T-nos

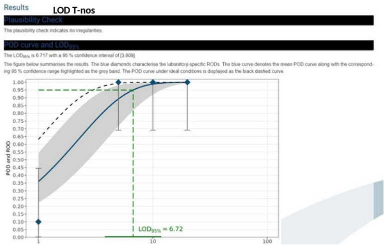

LOD PFMV

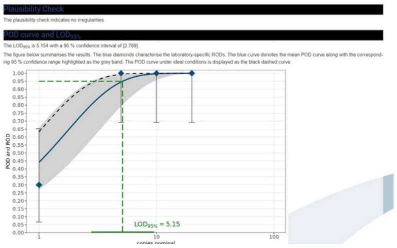

Eurofins GeneScan (Method 2)

LOD P35S

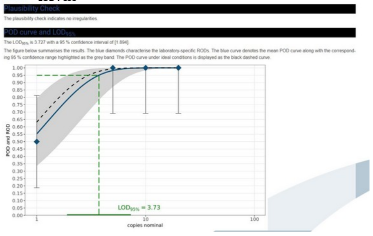

LOD T-NOS

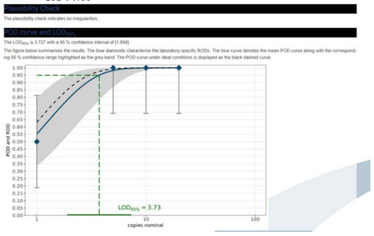

LOD PFMV

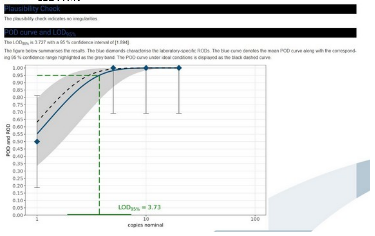

ThermoScientific (Method 3)

LOD P35S

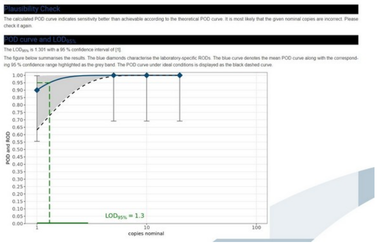

LOD T-nos

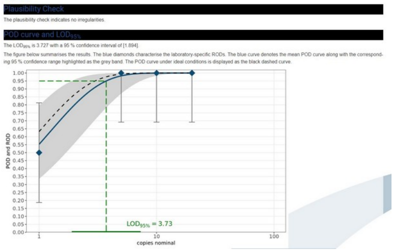

LOD PFMV

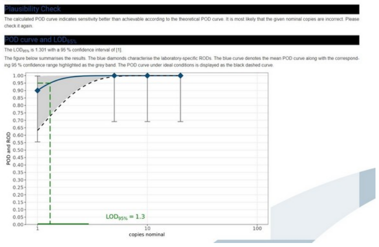

Italian NRL methods (Method 4)

LOD P35S

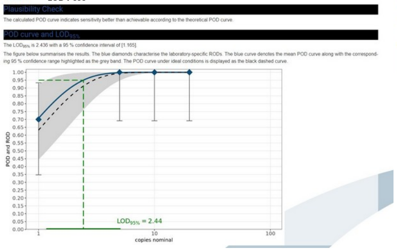

LOD T-nos

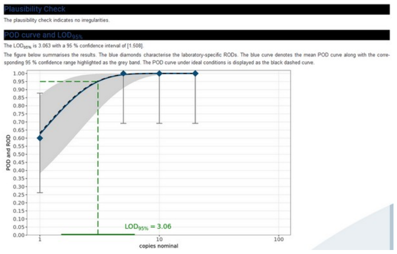

LOD PFMV

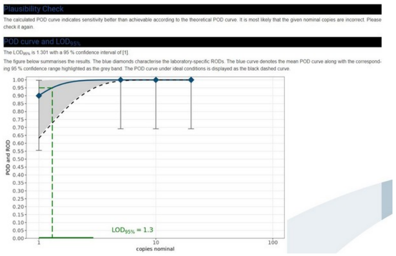

$LOD_{95\%}$ values verified by using Quodata web application

Screening kit	LOD		
	P35S	T-NOS	PFMV
R-Biopharm (Method 1)	4.42	6.72	5.15
Eurofins GeneScan (Method 2)	3.73	3.73	3.73
ThermoScientific (Method 3)	1.3	3.73	1.3
Italian NRL methods (Method 4)	2.44	3.06	1.3
